# Developing internal medicine subspecialty fellows’ teaching skills: a needs assessment

**DOI:** 10.1186/s12909-018-1283-2

**Published:** 2018-09-24

**Authors:** Jakob I. McSparron, Grace C. Huang, Eli M. Miloslavsky

**Affiliations:** 1 Division of Pulmonary and Critical Care Medicine, University of Michigan, 3916 Taubman Center, 1500 East Medical Center Drive, Ann Arbor, MI 48109 USA; 2 Department of Medicine, Beth Israel Deaconess Medical Center, 330 Brookline Ave., Boston, MA 02215 USA; 3 Division of Rheumatology, Allergy and Immunology, Department of Medicine, Massachusetts General Hospital, Yawkey Center for Outpatient Care, 55 Fruit Street, Suite 2C, Boston, MA 02114 USA

## Abstract

**Background:**

For academic physicians, teaching represents an essential skill. The proliferation of educator training programs aimed at residents and medical students signals the increasing commitment of training programs to develop teaching skills in their trainees as early as possible. However, clinical fellowships represent an important opportunity to advance training as educators. In addition to enriching the pipeline of future teachers, developing fellows as teachers augments the training experience for more junior trainees and may impact patient care. Fellows’ needs for programs to improve teaching skills have been largely unexplored.

**Methods:**

We conducted a multi-institutional needs assessment of internal medicine (IM) subspecialty fellows to gauge interest in teaching and improvement of teaching skills. We surveyed IM subspecialty fellows at three academic medical centers about their access to fellow-as-teacher programs and other mechanisms to improve their teaching skills during fellowship. We also elicited their attitudes towards teaching and interest in training related to teaching skills.

**Results:**

One hundred eighty-three fellows representing 20 programs and nine different subspecialties responded to the survey (48% response rate). The majority of participants (67%) reported having no specific training focused on teaching skills and only 12% reported receiving regular feedback about their teaching during their fellowship. Seventy-nine percent of fellows anticipated teaching to be part of their careers, and 22% planned to participate in medical education scholarship. Fellows reported a strong interest in teaching and programs aimed at improving their teaching skills.

**Conclusions:**

The majority of fellows reported a lack of mechanisms to advance their teaching skills as fellows, despite anticipating teaching to be an important aspect of their future careers and having strong interest in such programs. Our findings at three academic medical centers confirm a lost opportunity among subspecialty fellowships to accelerate teaching skills development for future educators.

**Electronic supplementary material:**

The online version of this article (10.1186/s12909-018-1283-2) contains supplementary material, which is available to authorized users.

## Background

Increasing availability of educator training programs, including those designed for trainees, supports the premise that skill development should start as early as possible in order to enhance the important role of physicians as teachers [1–3]. While resident as teacher programs are now widely available, relatively few programs have focused on improving fellows’ (subspecialty registrars’) teaching skills [4–7].

Enhancing fellows’ teaching skills may have broad-reaching advantages [8]. Improving teaching skills of trainees may have a significant impact on the learners with whom they interact. Patient care may benefit from increased communication between teams that occurs when fellows teach residents in the setting of consultation. Fellows may improve their own clinical skills and medical knowledge through teaching. Finally, fellows may positively influence career development of more junior trainees [9].

Little is known about Internal Medicine subspecialty fellows’ needs for programs that develop their skills in teaching [10]. Therefore, the purpose of our work was to assess the needs among Internal Medicine subspecialty fellows at three large academic medical centers toward career development as educators, specifically their attitudes towards teaching, improvement of their teaching skills and interest in pursuing careers in medical education.

## Methods

### Setting and participants

We surveyed Internal Medicine subspecialty fellows from 9 subspecialties in three large academic medical centers: Massachusetts General Hospital (MGH), Brigham and Women’s Hospital (BWH) and Beth Israel Deaconess Medical Center (BIDMC) all in Boston, MA. Subspecialty fellows are trainees who have completed general internal medicine training and are pursuing further training in a medical subspecialty, similar to a subspecialty registrar in the United Kingdom. Completion of the survey enabled participants to be entered in a drawing for an Apple iPad®. The study was approved by the Partners Institutional Review Board as exempt.

### Survey development

We created the survey by adapting questions from previous studies of attitudes towards teaching [10] and teaching self-efficacy [11] or developing questions based on prior qualitative research [12] (Additional file 1). The six domains we explored were: 1) attitudes towards teaching; 2) anticipated career path; 3) interest in teaching skills training during fellowship; 4) prior teaching experience and availability of teacher training in fellowship; 5) self-assessment of teaching skills; and 6) optimal content and format of teacher training programs for fellows [10–12]. We refined the final survey instrument through a collaborative, iterative review process including cognitive interviews and piloting with two Internal Medicine residents and two medical education experts with significant experience in survey design.

### Statistical analysis

We tabulated results using descriptive statistics. JMP Pro 12 (Cary, NC) was used for analysis.

## Results

One hundred eighty-three fellows from 20 programs representing nine different subspecialties responded to the survey (48% response rate) (Table 1).

**Table 1:** – Characteristics of study participants

Participating fellows	N (%)
Post Graduate Year (PGY)	
PGY-3	5 (3%)
PGY-4	40 (28%)
PGY-5	38 (27%)
PGY-6	30 (21%)
PGY-7	21 (15%)
PGY-8	9 (6%)
Fellowship	
Cardiology	40 (22%)
Endocrinology	11 (6%)
Gastroenterology	22 (12%)
Hematology/Oncology	30 (16%)
Infectious disease	17 (9%)
Nephrology	17 (9%)
Palliative Care	4 (2%)
Pulmonary / Critical Care Medicine	23 (13%)
Rheumatology	16 (9%)
Other	3 (2%)
Anticipated career activities	
Academics	156 (86%)
Not sure	17 (9%)
Private practice	9 (5%)
Basic science research	63 (35%)
Clinical research	125 (69%)
Patient care	154 (85%)
Teaching	144 (79%)
Education scholarship	40 (22%)
Administration	45 (25%)

### Attitudes towards teaching and anticipated career path

Fellows reported a strong interest in teaching medical students and residents (Table 2). The majority of fellows anticipated pursuing careers in academic medicine (86%), 79% felt that teaching would be a part of their career and 22% anticipated engaging in medical education scholarship, which was defined as curriculum development or medical education research (Table 1).

**Table 2:** – Fellow attitudes about teaching

	Strongly Disagree	Disagree	Neutral	Agree	Strongly Agree
I enjoy teaching residents and medical students	3 (2%)	0 (0%)	8 (5%)	45 (25%)	121 (68%)
If I had more time I would do more teaching	2 (1%)	2 (1%)	5 (3%)	60 (34%)	107 (61%)
Teaching residents is one of the responsibilities of a fellow	3 (2%)	0 (0%)	6 (3%)	58 (33%)	111 (62%)
Teaching medical students is one of the responsibilities of a fellow	3 (2%)	2 (1%)	15 (8%)	72 (40%)	86 (48%)

### Interest in and availability of teacher training

The majority of fellows reported a strong interest in improving their teaching skills (Table 3). Most fellows (88%) had formal teaching experiences prior to starting fellowship (e.g. teaching assistant, leading student or resident didactics) (Table 4). Sixty-seven percent of respondents reported having participated in teaching training during residency. However, only a minority of fellows (33%) reported participating in such training during fellowship. Twenty-nine percent of fellows reported having their teaching observed during fellowship and only 12% reported receiving feedback on their teaching at least once monthly.

**Table 3:** – Fellow attitudes towards teacher training

	Strongly Disagree	Disagree	Neutral	Agree	Strongly Agree
My teaching skills can be improved	2 (1%)	1 (1%)	10 (6%)	63 (35%)	102 (57%)
I am interested in receiving training to improve my teaching skills	5 (3%)	10 (6%)	29 (16%)	67 (38%)	66 (37%)
I want to receive more feedback about my teaching	2 (1%)	9 (5%)	35 (20%)	68 (38%)	64 (36%)

**Table 4:** – Prior teaching experience and teacher training

	N (%)
Teaching experience prior to fellowship	
Teaching during college or graduate school	101 (56%)
Formal teaching of medical students or residents	52 (29%)
Full time non-medical teaching	7 (4%)
No formal teaching experience	21 (12%)
Have you had training in education during residency?	
Yes	120 (67%)
No	59 (33%)
Have you had training in education during fellowship?	
Yes	59 (33%)
No	122 (67%)
Have you had observed teaching experiences during fellowship?	
Yes	51 (29%)
No	127 (71%)
How often have you received feedback on teaching during fellowship?	
Feedback on teaching (never or less than once monthly)	158 (88%)
At least once per month	21 (12%)

### Self assessment of teaching skills

Most fellows reported confidence in their ability to teach effectively across a range of teaching settings (teaching in the setting of consultation, giving a lecture, teaching in a group setting) (Table 5). Fellows reported the least confidence in learner-centered elements of teaching (e.g. learner assessment and feedback) and teaching within time constraints in the setting of consultation.

**Table 5:** – Fellow perceptions of their teaching skills

Skill	Definitely cannot	Probably cannot	Neutral	Probably can	Definitely can
*Teaching on the consult service*					
Can you figure out how much the intern already knows about the disease?	(0%)	13 (7%)	44 (25%)	102 (58%)	18 (10%)
Can you identify the major teaching points for this case?	1 (1%)	1 (1%)	7 (4%)	100 (56%)	68 (38%)
Can you teach effectively within the time constraints of a busy service?	1 (1%)	26 (15%)	47 (27%)	73 (41%)	30 (17%)
Can you give feedback to the intern about his/her approach to the patient thus far?	2 (1%)	17 (10%)	29 (16%)	99 (56%)	30 (17%)
*Giving a lecture to medical students*					
None of the students are planning to enter your sub-specialty field. Can you convey the importance of your topic to the students’ clinical training?	(0%)	(0%)	9 (5%)	108 (61%)	60 (34%)
This is the second block rotation for the 3rd year students. Can you accommodate the students’ differing clinical experiences?	(0%)	3 (2%)	35 (20%)	106 (60%)	33 (19%)
Can you address the different learning styles of your students? (e.g. quiet learner, dominant learner, etc...)	(0%)	21 (12%)	64 (36%)	69 (39%)	22 (13%)
*Teaching in resident case conference*					
The disease presented is very rare. Can you take the disease-specific elements and generalize them to broader principles?	(0%)	5 (3%)	24 (14%)	111 (63%)	35 (20%)
Some of the residents never speak up at conferences. Can you encourage participation from the quieter members?	1 (1%)	30 (17%)	53 (30%)	73 (42%)	18 (10%)
A resident asks you a question that is unrelated to your dedicated topic. Can you keep the discussion focused on your key teaching points?	(0%)	4 (2%)	42 (24%)	104 (59%)	25 (14%)
The audience includes all PGY levels. Can you address the different levels of the residents?	(0%)	6 (3%)	43 (25%)	101 (58%)	24 (14%)
You want to avoid asking questions that only test the recall of facts. Can you devise questions that evaluate your learners’ ability to apply their knowledge to a clinical situation?	(0%)	8 (5%)	47 (27%)	92 (53%)	28 (16%)

### Content and format of teacher training programs

One hundred and twenty fellows responded to a free-text question asking for their views on the content and format of curricula to improve fellow teaching skills that would be useful during fellowship. Table 6 summarizes key themes with representative responses. Workshops centering on key topics in medical education (e.g. giving feedback, delivering an effective lecture, small group teaching, asking effective questions, adult learning principles) and direct observation of teaching were the most common suggestions. Fellows also suggested that time and opportunities for teaching (in particular observed teaching) be allocated within the fellowship structure. Finally, several fellows expressed the view that simply emphasizing the importance of teaching within fellowship is an important step in promoting teaching during this phase of training. As one fellow stated, “just having it made clear that we are expected to teach residents, and being encouraged to do so by our attendings, would be a huge step.”

**Table 6:** Thematic analysis of preferred content and format of curricula to improve teaching skills

Theme	Examples
Desire for increased time and opportunities for teaching	“Time is the limiting factor. Give fellows and house staff more time if teaching is truly a priority.”
	“More time during fellowship to do this. More opportunities for formal teaching of residents and students if interested and would need coverage given to fellows for participation in these events. This can’t just be something added on.”
Emphasizing teaching as an important aspect of fellowship	“To be honest, just having it made clear that we are expected to teach residents, and being encouraged to do so by our attendings, would be a huge step.”
	“The reality is that everyone is busy all the time. So, if we fall back on this excuse, then no teaching will ever happen. It therefore behooves us to carve out dedicated time for teaching and learning…and recognize that, even though we are all busy, these twin missions are integral to our success as an institution and as a profession.”
Preference for workshop as format	“I would institute a work-shop like teaching environment. Fellows from one discipline should have the opportunity to interact with fellows across all disciplines (exchange ideas, troubleshoot common problems, etc.…).”
	“Workshops on teaching are great, especially since it is some protected time to think and reflect about teaching.”
Importance of adult learning theory as topic	“Some adult learning theory and how to ask higher order questions would be useful. My teaching experience is all just on the go.”
	“Adult learning theory seems most interesting to me.”
Importance of observed teaching	“We could discuss teaching as a goal with our consult attendings, and devise a system and identify a day or time when they rely on us to do the teaching in rounds and observe us- often in rounds with residents the fellow and attending both try to teach at the same time which makes it less productive.”
	“Direct observation of teaching with feedback is the most important.”
Desire to improve small group and lecture skills	“Specific lessons for large group teaching versus small group teaching would be helpful.”
	“I would have a teaching workshop that is also designed to teach how to give a great power point presentation.”

## Discussion

We examined Internal Medicine subspecialty fellows’ needs for teaching skills at three large academic medical centers. We demonstrated that in our sample, where the majority planned to pursue careers in academic medicine, fellows had a positive view of teaching and were interested in training to improve their teaching skills. Most fellows anticipated being involved in teaching during their careers and nearly a quarter planned to engage in medical education scholarship. Yet a minority of fellows reported access to such programs or regular feedback on their teaching during fellowship.

The lack of opportunities for fellows to develop as teachers is at odds with the multitude of resident-as-teacher programs available. Several factors may contribute. While it is widely recognized that residents do a large share of teaching students and junior residents, the role of fellows as teachers may be underrecognized. However, teaching has been identified as an important component of consultation [13], which constitutes a large part of fellowship clinical training. Fellow teaching in this setting may have a broad reaching positive impact, highlighting the important role that fellows have as teachers [8, 9, 12]. Another contributing factor may be that fellows are felt to have well-developed teaching skills from prior experiences and resident-as-teacher training during residency. This is in part supported by the high level of confidence fellows reported in their teaching skills. However, teaching as a fellow may differ from teaching as a resident. The majority of fellows’ teaching opportunities occur in the setting of consultation which differs qualitatively from teaching experiences fellows may have previously had; common challenges in teaching are especially accentuated by consultation, such as time constraints, interacting with groups of learners at different levels, and teaching learners unfamiliar topics [12, 14]. Indeed, fellowship training may itself help fellows recognize the need for additional training. Finally, fellows have considerable demands on their time. Learning a new specialty, providing clinical care and engaging in research are all important priorities. However, given that fellows are our future faculty and the strong interest in improving teaching skills demonstrated in our study, enhancing fellows’ teaching skills is of import. In addition, given that a considerable proportion of fellows plan to pursue educational scholarship during their careers, such programs may also contribute to developing fellows’ scholarly pursuits.

Few articles have focused on fellowship as an opportune timeframe to foster teaching skills. Richards et al. surveyed pulmonary/critical care fellows in a single program, demonstrating that 75% of fellows were interested in improving their teaching skills and approximately half were interested in careers in education [10]. Kelly et al. performed a similar study of respiratory specialist registrars in Ireland [15]. The majority of respondents (81%) were interested in pursuing careers as educators, and 85% were interested in additional education training. While we found similar levels of interest in additional education training, we expanded on this work by examining multiple specialties across several large academic medical centers and expanding the number of domains examined. Previous studies also suggest that the desire for further training is shared by fellowship program directors; subspecialty fellowship program directors feel that their trainees would benefit from increased formal education related to teaching skills [7, 16].

Our study has several limitations. We studied three academic institutions in a single geographic area where the vast majority of fellows plan to remain in academic medicine, which limits the generalizability of our findings. Approximately half of the fellows completed the survey, which may have selected for individuals specifically interested in teaching. We were not able to compare the survey data to objective measures of fellow’s teaching skills, nor were we able to determine fellows’ actual career paths after fellowship completion. These remain important areas for future study.

## Conclusion

Our study provides evidence that a gap exists between fellows’ needs for development as educators and mechanisms to grow those skills. Efforts to develop and implement programs to improve fellow teaching skills and provide further support for fellows interested in careers in medical education are warranted. Future studies should focus on the impact of such programs on fellows’ teaching skill and career development, resident and medical student learning and patient care.

## Additional file

Additional file 1: Survey instrument. This file is the survey which was used in this study. (DOCX 27 kb)
