# Supplementary material for: Developing internal medicine subspecialty fellows’ teaching skills: a needs assessment
Source: BMC Med Educ. 2018 Sep 24;18:221. doi: 10.1186/s12909-018-1283-2 (PMC6154890; doi:10.1186/s12909-018-1283-2)
Supplement: Supplementary file 1 — Survey instrument. This file is the survey which was used in this study. (DOCX 27 kb) [file 12909_2018_1283_MOESM1_ESM.docx]

Fellow survey instrument

Thank you for completing this survey. The purpose of this survey is to examine your attitudes and experiences teaching residents and medical students.   DEFINITION: For the purposes of this survey, "teaching" includes didactic lectures/small groups as well as informal interactions in the hospital where you are conveying information to a trainee. The entire survey should take 5 minutes to complete.    Important: Your responses will not be linked to any identifiable information.  The data will be used for research purposes only and your responses will not be part of any formal evaluation. You can exit the survey at any time and your responses up to that point will be saved; however, once you exit you cannot go back to complete the survey, so please try to finish in one sitting. Please use the forward arrow button in the bottom right of each page to move forward in the survey.   If you have any questions or concerns about the survey, please contact Dr. Eli Miloslavsky (emiloslavsky@mgh.harvard.edu) or Dr. Alberto Puig (apuig@mgh.harvard.edu)   If you would like to be entered in a drawing for an iPad Air (or gift certificate for equivalent value), please enter your email address in the textbox below (your email will not be linked to your responses).  One prize will be raffled off among the survey respondents.

________________________________________________________________

What is your level of training?

- PGY3 (1)
- PGY4 (2)
- PGY5 (3)
- PGY6 (4)
- PGY7 (5)
- PGY8 (6)

What is your sub-specialty?

- Allergy (1)
- Cardiology (including interventional, electrophysiology) (2)
- Pulmonary / Critical Care Medicine (3)
- Endocrinology (4)
- Gastroenterology (5)
- Hematology/Oncology (6)
- Infectious disease (7)
- Nephrology (8)
- Rheumatology (9)
- Palliative Care (10)
- Other (11) ________________________________________________

Do you plan to pursue private practice or academics?

- Private practice (1)
- Academics (2)
- Not sure (3)

Which of the following activities do you plan to include in your career? (Check all that apply)

- Basic science research (1)
- Clinical research (2)
- Patient care (3)
- Teaching (4)
- Medical education scholarship (curriculum development, research, etc) (5)
- Administration (6)
- Not sure (7)

What prior formal teaching experience do you have (not including informal teaching on the wards)? *(check all that apply)*

- Full-time non-medical teaching in the past (school teacher, Teach for America, etc...) (1)
- Teaching during college/graduate school (TA for class, standardized test instructor, etc...) (2)
- Formal teaching of medical students/residents (formal lectures, tutorial leader,etc...) (3)
- Other (please specify) (4) ________________________________________________
- No formal prior teaching experience (5)

During your RESIDENCY, what training did you have in teaching or education?  (Check all that apply)

- None (1)
- Lectures/didactic sessions/workshops dedicated to teaching (2)
- Formal coursework (e.g. education fellowship, master’s degree) (3)
- Other (please specify) (4) ________________________________________________

During your FELLOWSHIP, what training have you had in teaching or education?  (Check all that apply)

- None (1)
- Lectures/didactic sessions/workshops dedicated to teaching (2)
- Formal coursework (e.g. education fellowship, master’s degree) (3)
- Other (please specify) (4) ________________________________________________

During FELLOWSHIP, has your teaching ever been observed by a faculty member or peer specifically for the purpose of providing feedback on your teaching skills?

- Yes (1)
- No (2)

On average, how frequently has your teaching been formally observed for the purpose of providing feedback about your teaching?

- Less than 1 time per year (1)
- 1-2 times per year (2)
- 3-4 times per year (3)
- 5 or more times per year (4)

During a typical month on clinical service, how frequently do you receive specific feedback about your teaching from a faculty member or another fellow (that is, comments about specific teaching tactics or areas for improvement, not simply "good job")?

- Never (1)
- Rarely, fewer than once a month (2)
- 1-2 times per month (3)
- 3-4 times per month (4)
- More than 5 times per month (5)

With regard to your own teaching skills and receiving feedback about your teaching, to what extent do you agree with the following statements?

|  | Strongly Disagree (1) | Somewhat Disagree (2) | Neutral (3) | Somewhat Agree (4) | Strongly Agree (5) |
| --- | --- | --- | --- | --- | --- |
| I want to receive more feedback about my teaching (1) |  |  |  |  |  |
| I enjoy teaching residents and medical students (2) |  |  |  |  |  |
| My teaching skills can be improved (3) |  |  |  |  |  |
| I am interested in receiving training to improve my teaching skills (4) |  |  |  |  |  |

Thinking about teaching residents and medical students at your institution, to what extent do you agree with the following statements?

|  | Strongly disagree (1) | Disagree (2) | Neutral (3) | Agree (4) | Strongly Agree (5) |
| --- | --- | --- | --- | --- | --- |
| Teaching residents is one of the responsibilities of a fellow (1) |  |  |  |  |  |
| Teaching medical students is one of the responsibilities of a fellow (2) |  |  |  |  |  |
| If I had more time I would do more teaching (3) |  |  |  |  |  |

| Page Break |  |
| --- | --- |

During your teaching interactions with residents and students, what IMPACT does each of the following potential barriers have on the encounter?

|  | None at all (1) | A little bit (2) | A moderate amount (3) | A lot (4) | Major impact (5) |
| --- | --- | --- | --- | --- | --- |
| Resident too busy to learn (1) |  |  |  |  |  |
| Frequent resident pass-offs (2) |  |  |  |  |  |
| Resident not knowing the patient well (3) |  |  |  |  |  |
| Locating the responding resident when on the floor (4) |  |  |  |  |  |
| Resident does not seem interested in learning (5) |  |  |  |  |  |
| Fellow not knowing resident (6) |  |  |  |  |  |
| Fellow too busy to teach (7) |  |  |  |  |  |
| Fellow teaching not encouraged by division/attendings (8) |  |  |  |  |  |
| Fellow not receiving positive reenforcement on their teaching from residents or attendings (9) |  |  |  |  |  |

The following questions relate to teaching scenarios you may encounter as a fellow.  Please comment on your confidence level in reaching the objectives described below

You come to the floor to meet with an internal medicine intern about a complicated case you are seeing on the consult service.  You plan to spend no more than five to seven minutes discussing the case and conveying your recommendations.  You would also like to teach the intern about this case.

|  | Definitely cannot (1) | Probably cannot (2) | Neutral (3) | Probably can (4) | Definitely can (5) |
| --- | --- | --- | --- | --- | --- |
| Can you figure out how much the intern already knows about the disease? (1) |  |  |  |  |  |
| Can you identify the major teaching points for this case? (2) |  |  |  |  |  |
| Can you teach effectively within the time constraints of a busy service? (3) |  |  |  |  |  |
| Can you give feedback to the intern about his/her approach to the patient thus far? (4) |  |  |  |  |  |

| Page Break |  |
| --- | --- |

You are scheduled to give a monthly talk to a group of four 3rd year medical students rotating in your sub-specialty.

|  | Definitely cannot (1) | Probably cannot (2) | Neutral (3) | Probably can (4) | Definitely can (5) |
| --- | --- | --- | --- | --- | --- |
| None of the students are planning to enter your sub-specialty field. Can you convey the importance of your topic to the students’ clinical training? (1) |  |  |  |  |  |
| This is the second block rotation for the 3rd year students. Can you accommodate the students’ differing clinical experiences? (2) |  |  |  |  |  |
| Can you address the different learning styles of your students? (e.g. quiet learner, dominant learner, etc...) (3) |  |  |  |  |  |

You are leading Friday morning resident case conference and will give a short presentation related to a disease in your sub-specialty.

|  | Definitely cannot (1) | Probably cannot (2) | Neutral (3) | Probably can (4) | Definitely can (5) |
| --- | --- | --- | --- | --- | --- |
| The disease presented is very rare. Can you take the disease-specific elements and generalize them to broader principles? (1) |  |  |  |  |  |
| Some of the residents never speak up at conferences. Can you encourage participation from the quieter members? (2) |  |  |  |  |  |
| A resident asks you a question that is unrelated to your dedicated topic. Can you keep the discussion focused on your key teaching points? (3) |  |  |  |  |  |
| The audience includes all PGY levels. Can you address the different levels of the residents? (4) |  |  |  |  |  |
| You want to avoid asking questions that only test the recall of facts. Can you devise questions that evaluate your learners’ ability to apply their knowledge to a clinical situation? (5) |  |  |  |  |  |

| Page Break |  |
| --- | --- |

If a curriculum was designed to improve fellow teaching skills, what suggestions do you have for the format and/or specific content? (examples of format include: workshop, multiple seminars, direct observation of teaching, etc.;  examples of content include: feedback, asking higher order questions, adult learning theory, etc.)

________________________________________________________________

________________________________________________________________

________________________________________________________________

________________________________________________________________

________________________________________________________________
